# *Drosophila* Zpr1 (Zinc Finger Protein 1) Is Required Downstream of Both EGFR And FGFR Signaling in Tracheal Subcellular Lumen Formation

**DOI:** 10.1371/journal.pone.0045649

**Published:** 2012-09-18

**Authors:** Oscar E. Ruiz, Linda S. Nikolova, Mark M. Metzstein

**Affiliations:** Department of Human Genetics, University of Utah, Salt Lake City, Utah, United States of America; University of Massachusetts Medical School, United States of America

## Abstract

The cellular and molecular cues involved in creating branched tubular networks that transport liquids or gases throughout an organism are not well understood. To identify factors required in branching and lumen formation of *Drosophila* tracheal terminal cells, a model for branched tubular networks, we performed a forward genetic-mosaic screen to isolate mutations affecting these processes. From this screen, we have identified the first *Drosophila* mutation in the gene *Zpr1* (*Zinc finger protein 1*) by the inability of *Zpr1*-mutant terminal cells to form functional, gas-filled lumens. We show that *Zpr1* defective cells initiate lumen formation, but are blocked from completing the maturation required for gas filling. Zpr1 is an evolutionarily conserved protein first identified in mammalian cells as a factor that binds the intracellular domain of the unactivated epidermal growth factor receptor (EGFR). We show that down-regulation of EGFR in terminal cells phenocopies *Zpr1* mutations and that *Zpr1* is epistatic to ectopic lumen formation driven by EGFR overexpression. However, while *Zpr1* mutants are fully penetrant, defects observed when reducing EGFR activity are only partially penetrant. These results suggest that a distinct pathway operating in parallel to the EGFR pathway contributes to lumen formation, and this pathway is also dependent on Zpr1. We provide evidence that this alternative pathway may involve fibroblast growth factor receptor (FGFR) signaling. We suggest a model in which Zpr1 mediates both EGFR and FGFR signal transduction cascades required for lumen formation in terminal cells. To our knowledge, this is the first genetic evidence placing Zpr1 downstream of EGFR signaling, and the first time Zpr1 has been implicated in FGFR signaling. Finally, we show that down-regulation of Smn, a protein known to interact with Zpr1 in mammalian cells, shows defects similar to *Zpr1* mutants.

## Introduction

Branched tubular networks, such as the vascular (blood) and respiratory systems, are a common structural design used to facilitate the transport of liquids and gases throughout the body. The cellular cues and signaling required for generating these complex networks, and tailoring each structure for a specific need (e.g., transporting liquid vs. transporting gases) are not well understood. To identify components involved in tubular network formation we are studying the *Drosophila melanogaster* larval tracheal (respiratory) system. The larval tracheal system is composed of a network of approximately 10,000 interconnected tubes that serve to transport oxygen and other gases throughout the body [1], [2]. To construct this elaborate tubular network, cells within the tracheal epithelium first undergo a series of coordinated and stereotyped branching and tubulogenesis events during mid-embryogenesis. These events are primarily regulated by a fibroblast growth factor ligand (FGF) and receptor (FGFR), encoded by the *branchless* (*bnl*) and *breathless* (*btl*) genes, respectively [3], [4]. These Bnl- and Btl-mediated early outgrowth events provide a cellular framework for what will eventually be the larval tracheal system. The mature larval tracheal system can be subdivided into three morphologically distinct tube types [5]. The first type are multicellular structures in which polarized sheets of epithelial cells are shaped to create tubes [6], [7]. Cross section of such tubes reveals intercellular junctions that maintain a permeability barrier for the lumen, the hollow space through which gases are transported. The second type of tubes are unicellular and are made from single cells that wrap around their long axis and form an autocellular junction [8]. The third type of tracheal tubes form in specific cell type called terminal cells [9], [10]. Terminal cells are characterized by their extensive subcellular branching and these branches contain completely subcellular lumens, which form without cell junctions [1], [9]. In contrast to the stereotyped branching and lumen formation observed during early tracheal morphogenesis, terminal cell branch outgrowth is a dynamic process and is controlled by the extent of hypoxia in target tissues and in the terminal cells [11], [12]. Thus, terminal cells may provide a model for understanding the development of fine capillaries within the vertebrate vasculature whose development also depends on tissue oxygen status [13].

To identify factors required for branching and lumen formation specifically in terminal cells, we have analyzed mutants obtained from a forward genetic-mosaic screen of the *Drosophila* X chromosome. Such an approach on other *Drosophila* chromosomes has proven successful at identifying genes affecting diverse aspects of terminal cell development [14], [15]. We show here that one of the alleles identified in our screen is the first *Drosophila* mutation of the *Zpr1* (*Zinc finger protein 1*) gene. Zpr1 is an evolutionarily conserved protein characterized by two C4 zinc fingers and two Zpr1-specific conserved homology domains [16]. Zpr1 was first identified in mammalian cells as a cytoplasmic protein that was capable of binding the intracellular domain of the unactivated EGF receptor (EGFR) [16]. Here we report that Zpr1 is required for terminal cell lumen maturation and show that Zpr1 function lies downstream of both the FGFR and the EGFR in regulating this process. Finally, we show that the Zpr1 interacting protein SMN (survival motor neurons) [17] is required for terminal cell lumen development, suggesting a developmental role for the evolutionarily conserved Zpr1/SMN pathway.

## Results

### A mutant required for branching and lumen formation in *Drosophila* terminal tracheal cells

**Figure 1:**
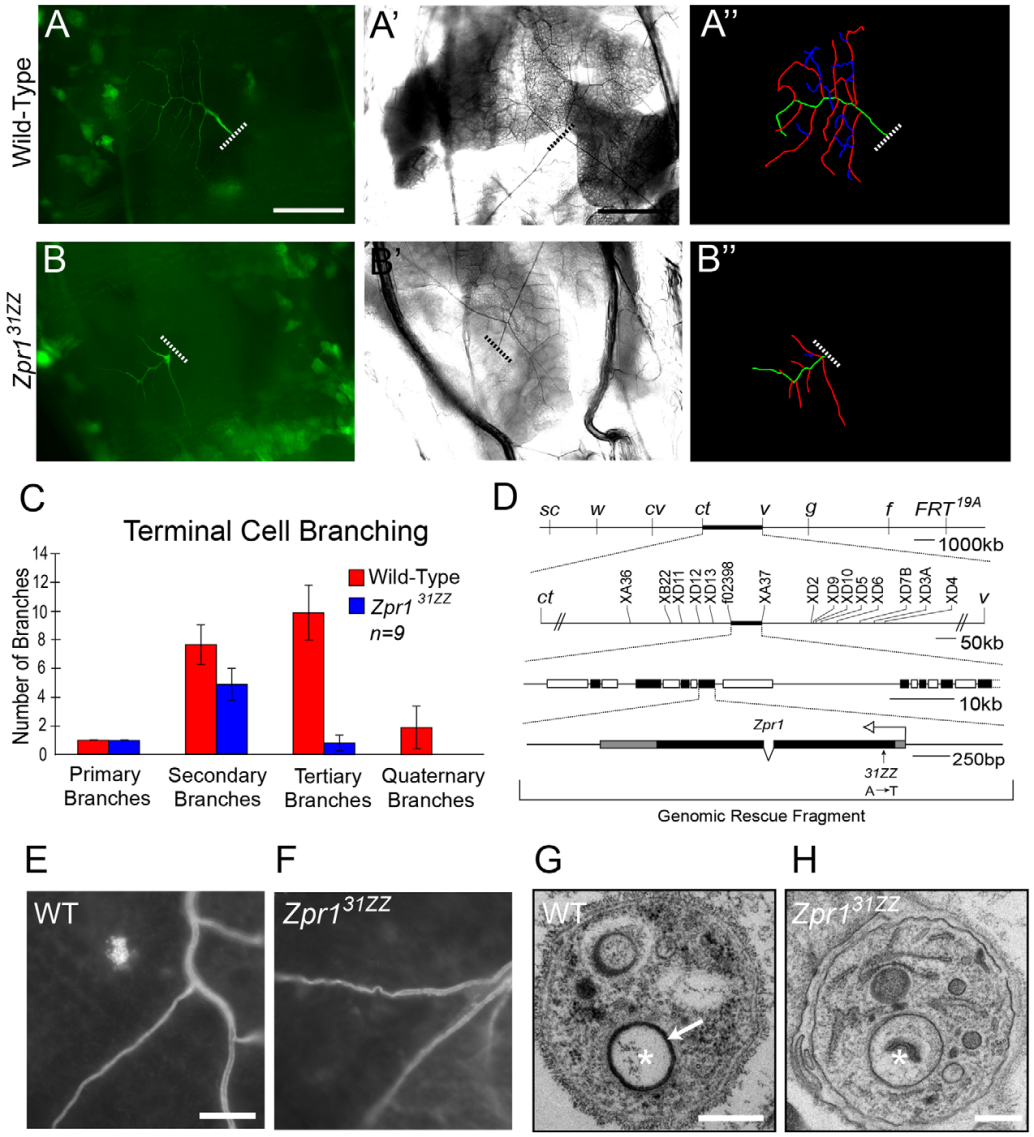
*Zpr1* is required for branching and lumen maturation in terminal cells. Mosaic L3 larvae generated using the MARCM system with homozygous mutant cells marked by the expression of GFP. Tracheal cell branching and gas-filling patterns are characterized by using GFP (A, B) and brightfield microscopy (A', B'), respectively. (A'', B'') Tracing of the branching patterns of cells shown in A and B. Wild-type terminal cells undergo extensive branching and have a single air-filled lumen in each branch (A–A''). *Zpr1* homozygous mutant cells exhibit branching defects and fail to generate a gas-filled lumen (B–B''). Dashed lines indicate proximal end of the cell. (C) Branching was quantified by designating branches of terminal cells as primary (green), secondary (red), tertiary (blue), or quaternary branch and totaling all branches. Tertiary and quaternary branching is affected in *Zpr1* mutants. Error bars are ±2 S.E.M of each class (*n* = 9). (D) Mapping and identification of *31ZZ* mutant. Genetic map of the X chromosome and the *ct*-*v* interval. A combination of visible markers, single nucleotide polymorphisms (XA, etc.) and transposon markers were used to map the lethality associated with *31ZZ* to a 72 kb interval. In the middle panel, black and white boxes represent individual genes within the 72 kb region. Subsequent sequencing of genes in the region identified a single A to T transversion at base position 172 of the *Zpr1* coding sequence. In the gene structure shown at the bottom, black boxes are *Zpr1* coding regions; shaded boxes, 5′ and 3′ untranslated regions; open arrow, direction of transcription; bracket: *Zpr1* genomic rescue construct. (E, F) Wild-type (E) and *Zpr1* mutant terminal branches (F) stain with the luminal marker α-Wkdpep. (G, H) TEM analysis of wild-type (G) and *Zpr1* mutant terminal branches (H). The lumens are marked with asterisks. The wild-type lumen is clear of cellular material while the *Zpr1* mutant lumen is obstructed. The wild-type lumen is lined with darkly staining cuticle (arrow) that is lacking in the *Zpr1* mutant lumen. Bars: A, 200 µm; E (and for F) 10 µm; G & H, 400 nm.

To identify genes involved in tracheal terminal cell development, a forward genetic-mosaic screen of lethal mutations on the X chromosome was performed by using the MARCM system [18] to generate positively (GFP) labeled homozygous mutant cells in heterozygous animals (M.M.M. and M.A. Krasnow, unpublished results). Homozygous mutant cells were then scored for tracheal branching and air-filling defects by using a combination of fluorescence microscopy and brightfield optics, to visualize terminal branching patterns and gas-filling respectively. Wild-type terminal cells have a single central terminal branch from which secondary branches sprout, subsequent branching occurs from these secondary branches to generate tertiary branches and from these form quaternary branches (Fig. 1A, A''). Wild-type cells also posses a single gas-filled lumen that runs through every branch class (Fig. 1A'). Among the 900 lines that were scored, a mutant designated *31ZZ* was identified by its complete failure to generate gas-filled lumens (Fig. 1B'). *31ZZ* mutant cells also show a significant branching defect (Fig. 1B, B''); we find that *31ZZ* mutants are able to generate a central branch and secondary branches, but fail to undergo tertiary and quaternary branching (Fig. 1C). While the branching phenotype was highly variable, gas-filling is completely abrogated in all *31ZZ* mutant terminal cells.

### *31ZZ* is an allele of *Drosophila Zpr1*

To identify the causative mutation in *31ZZ* we mapped the lethality to a discrete genetic interval on the X chromosome using a multipoint recombination mapping strategy (see Materials and Methods). This analysis placed the lethality associated with *31ZZ* to the 3.3 Mbp (megabase pair) interval between the *ct* and *v* genes. The interval containing *31ZZ* was then refined by using single nucleotide polymorphisms (SNP) and transposable element insertions as molecular markers of meiotic recombination events. This allowed us to map the *31ZZ* mutation to a 72 kb (kilobase) interval containing 15 genes (Fig. 1D). Sequencing of the genes in this region identified a single point mutation (an A to T transversion) in the first exon of the gene *Zpr1*. This mutation results in a premature termination codon at amino acid position 58. To confirm this mutation as the causative allele in *31ZZ*, a 3.2 kb transgene spanning the *Zpr1* genomic locus as well as 949 bp (base pairs) upstream of the gene was transformed into flies and tested for rescue of the lethality and gas-filling defect associated with *31ZZ*. We find that a single copy of this transgene is able to rescue the lethality associated with *31ZZ*, as well as all terminal cell defects (data not shown). Additionally, we generated transgenic flies carrying a *Zpr1* cDNA under the control of the GAL4/UAS system [19]. We found this construct was able to rescue the gas filling defect in 3*1ZZ* mutant cells when specifically expressed in terminal cells (data not shown). From these experiments we conclude that the mutation identified in *Zpr1* is responsible for the lethality and gas-filling defects associated with mutant line *31ZZ*.

### *Zpr1* is required for maturation of the terminal cell lumen

Using of bright-field microscopy to examine tracheal cells, we cannot distinguish between complete lack of lumen formation or a subsequent defect in gas-filling. To determine at which step *31ZZ* affects the process of lumenogenesis, we performed two analyses. First, we used an characterized antiserum (α-Wkdpep) that reacts against a component present in the terminal cell lumen [20], to stain *31ZZ* mutant cells. We found that *31ZZ* terminal cell branches labeled with this antisera (Fig. 1F), and the staining observed was very similar to that observed in wild-type cells (Fig. 1E), though occasionally portions of *31ZZ* branches, did not show staining. Second, we used transmission electron microscopy (TEM) to examine *31ZZ* mutant tracheal terminal branches. Because it is difficult to identifying mutant cells in mosaic animals when using TEM, we instead used RNAi to inactivate *Zpr1* throughout the tracheal system, so that all terminal cells in fixed samples should have the *31ZZ* defect. We first confirmed that *Zpr1* RNAi leads to gas-filling defects similar to *Zpr1^31ZZ^* mutant cells (Fig. S1A). We next examined these tracheal cells in these animals by TEM. In wild-type tracheal branches, the lumen appears as a membrane bound compartment, typically centrally placed within the branch. The inside of the luminal membrane is lined with a chitinous cuticle, arranged in rings known as taenidial folds [21], and the central space is clear of any cellular material (Fig. 1F). By contrast, we found that while *Zpr1* RNAi terminal branches contain an internal, membrane-bound structure, positioned similarly to a mature lumen, the mutant lumens lack a developed cuticle and the lumen is frequently occluded with staining material (Fig. 1G and Fig. S1B). While the level of gene activation by RNAi may not represent a complete loss of gene function, the conclusion from both our staining and EM analysis is that *Zpr1*-mutant terminal cells initiate lumen formation, but that progression to a mature, gas-filled lumen is blocked.

### Loss of EGFR function results in terminal cell gas-filling defects

**Figure 2:**
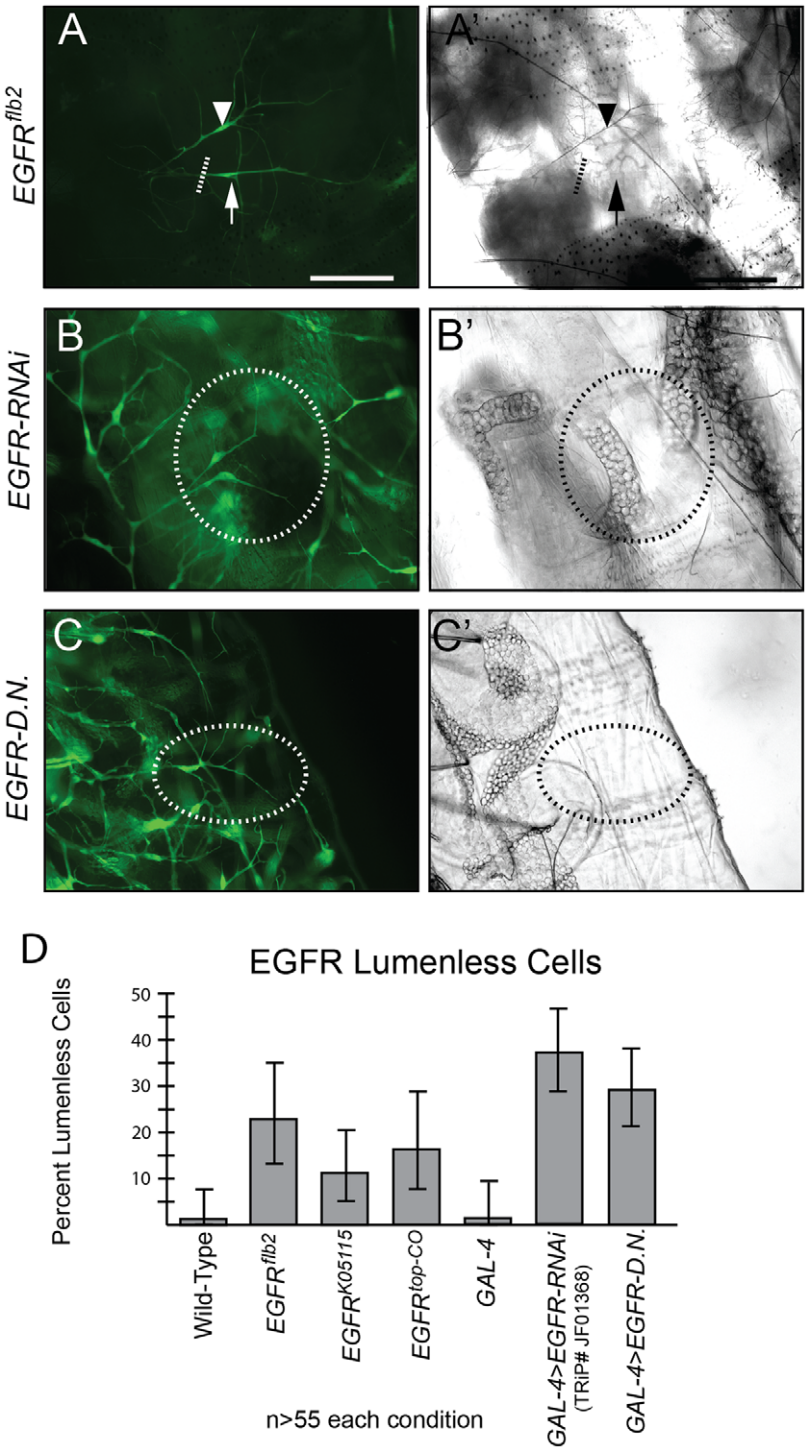
Loss of EGFR function in terminal cells results in a partially penetrant lumen gas-filling defect. (A) Two homozygous *EGFR^f2^* mutant cells, marked by the expression of GFP. One cell (arrow) shows a gas-filling defect, while the other cell (arrowhead) has an apparently normal, gas-filled lumen (brightfield image in A'), indicating the partial penetrance of the EGFR loss of function. Dashed line indicates proximal end of the non-filled mutant cell. Tracheal-specific expression of EGFR-RNAi (B) and a dominant negative EGFR (C) results in gas-filling defects (B', C'). Position of cells is indicated by dashed circles. (D) Quantification of penetrance of the EGFR loss-of-function phenotype. For all conditions quantified a minimum of 55 cells were scored. Error bars represent 95% confidence interval of the binomial distribution.

Zpr1 was first identified in a yeast 2-hybrid screen as a protein that was capable of binding to the intracellular domain of the unactivated EGF receptor [16]. Roles for EGFR signaling in the development [22], [23] and maintenance [24] of the *Drosophila* embryonic tracheal system have been previously characterized. In particular, over activity of the EGFR has been shown to be associated with an increase in embryonic lumen formation [22]. Therefore, we hypothesized that Zpr1 mediates an EGFR signaling cascade required for subcellular lumen formation or maturation. To explore this, we determined whether loss of EGFR activity had an effect on terminal cell lumenogenesis. First, we tested the effect of EGFR loss-of-function mutations on terminal cell development by examining homozygous mutant terminal cells in mosaic animals. We found mutations in any of three null alleles of the EGFR (*EGFR^K05115^, EGFR^f2^, EGFR^top-co^*) resulted in terminal cells without a visible gas-filled lumen (Fig. 2A, A′). In contrast to the robust phenotype of *Zpr1* mutants, in which all mutant cells are lack a gas-filled lumen, only a small percentage (around 15% after scoring at least 55 animals for each genotype) of terminal cells mutant for the EGFR have defective lumens (Fig. 2D). Interestingly, among terminal cells mutant for the *EGFR* that have the lumen defect, most display a branching defect similar to that observed in *Zpr1* mutant cells.

To determine whether the low penetrance of defects observed was a characteristic of using EGFR loss-of-function alleles, or a broader phenomenon of EGFR signaling, we conducted a series of experiments using different manipulations to reduce EGFR function. First, we downregulated the EGFR specifically in the tracheal system by using the tracheal specific *btl-GAL4* [25] driver to express an RNAi transgene directed against the EGFR. Similar to the loss-of-function alleles, we observed terminal cells with strong gas-filling defects (Fig. 2B, B'). Again, similar to the loss-of-function alleles, this defect was only partially penetrant (Fig. 2D). Other RNAi transgenes tested displayed similar low-penetrance terminal cell gas-filling defects when expressed in the tracheal system (data not shown). We next tested an EGFR dominant negative transgene (Fig. 2C, 2C'). This manipulation also led to gas-filling defects in terminal cells of a similar penetrance to the loss-of-function or RNAi experiment (Fig. 2D). Thus, multiple manipulations which reduce the EGFR in terminal cells result in a partially penetrant gas-filled lumen formation defect.

### EGFR overexpression results in ectopic lumen formation in a Zpr1-dependent manner

**Figure 3:**
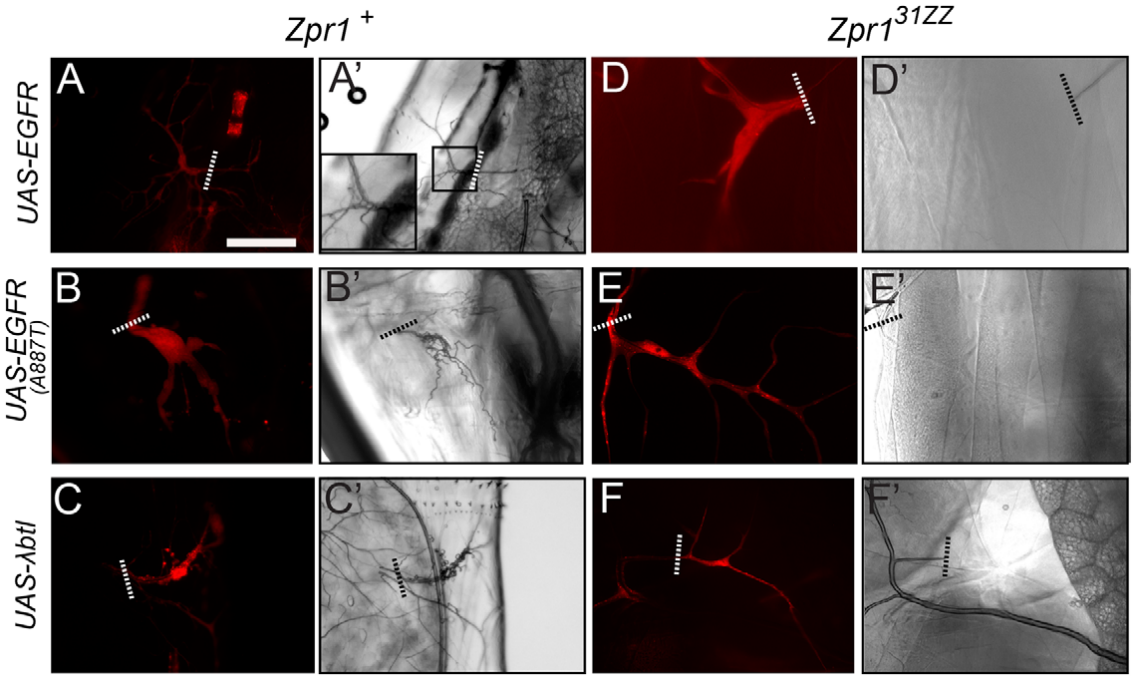
*Zpr1* is epistatic to both EGFR and FGFR signaling in terminal cells. Tracheal cell branching patterns and gas-filled lumens are characterized by using DsRed fluorescence (A–F) and brightfield microscopy (A'–F') respectively. Over-expression of wild-type (A) or activated (B) forms of the EGFR in terminal cells drives luminal overgrowth (A', B'. Inset in A' shows a close up of the cell body containing the extra lumens). Over-expression of an activated form of the FGF receptor in wild-type terminal cells results in extensive overgrowth of branches (C) and lumens (C'). *Zpr1* mutants are epistatic to the extensive luminal growth caused by the overexpression of wild-type EGFR (D, D'), activated EGFR (E, E') and activated FGFR (F, F'). Dashed lines indicate the proximal ends of the DsRed labeled cells. Bar, 100 µm for A, B, C, E, F; 50 µm for D.

Next, we tested if an increase in EGFR activity could induce lumen formation in tracheal terminal cells. We tested this by overexpressing wild-type and constitutively active versions of the EGFR. We found that over-expression of both the wild-type (Fig. 3A, A') and the activated EGFR (Fig. 3B, B') resulted in an increase in lumen formation, characterized by multiple air-filled lumens within a single cell. This effect is reminiscent of the result of increasing receptor tyrosine kinase (RTK) signaling in the embryonic tracheal system: this manipulation leads to ectopic embryonic lumen formation, though in embryos the defect is expressed as dilations of existing lumens, rather than generation of new lumens [22].

We next tested whether *Zpr1* lies downstream of the EGFR-mediated lumen formation by examining the epistatic relationship of *Zpr1* and the EGFR overexpression transgenes. We made MARCM mosaics in which *Zpr1* loss-of-function cells, positively marked by expression of a UAS-DsRed transgene, simultaneously overexpress EGFR. We found that *Zpr1* mutant cells that overexpress either the wild-type or the activated form of the EGFR lack gas-filled lumens, and appear essentially identical to *Zpr1* mutant cells (Fig. 3D, E). This finding suggests Zpr1 functions downstream of the EGFR, consistent with what was proposed from cell culture experiments [16]. These data provide the first *in vivo* evidence for functional relevancy of the EGFR-Zpr1 interaction and further indicate a role for EGF signaling in the regulation of lumen formation during development of terminal cells.

### Zpr1 lies downstream of multiple RTKs

The incomplete penetrance of our EGFR loss-of-function experiments led us to consider the possibility that other active RTKs could be feeding into the Zpr1 pathway. To test this, we examined the best studied RTK in the *Drosophila* tracheal system, the FGFR Btl [4]. We could not directly test loss of FGFR in lumen development because tracheal cells mutant for *btl* fail to outgrow or undergo branching [26]. To bypass this early Btl requirement, we tested the epistatic relationship of Zpr1 and FGFR by expressing an activated form of the Btl receptor (*λbtl*) [27]. We found that overexpression of this activated form of the FGFR in terminal cells resulted in increased lumen formation as visualized by multiple, gas-filled lumens within single cells (Fig. 3C, C'). To test whether loss of Zpr1 could suppress this FGFR mediated overgrowth, we made MARCM mosaics in which *Zpr1* loss-of-function cells simultaneously overexpress *λbtl*. We find that all *Zpr1* homozygous mutant cells overexpressing *λbtl* display a *Zpr1*-like phenotype (Fig. 3F, F'), indicating that Zpr1 is epistatic to FGFR overexpression in terminal cell lumen formation.

### *Drosophila* Smn is required for terminal cell lumen development

**Figure 4:**
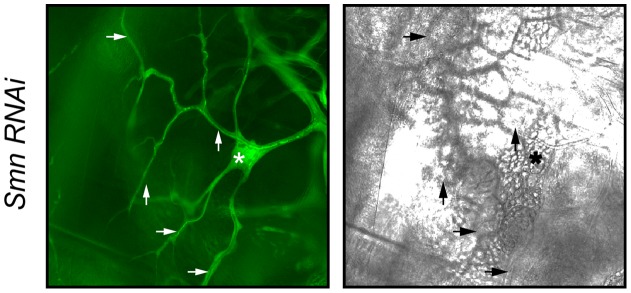
*Smn* is required for lumen formation and/or maturation in terminal cells. Expression of a RNAi transgene directed against *Smn* using the tracheal-specific driver *btl-GAL4* results in terminal cells lacking a gas-filled lumen, as observed using bright field microscopy (right), but with essentially normal branching (GFP image, left). Arrows indicate some of the branches to help demark the lack of gas filling; the asterisk indicates the cell body.

One known interactor with the Zpr1 protein in mammalian cells is the survival of motor neurons protein, SMN1 [17], [28]. This interaction is known to be required for SMN1 function [29]–[31]. To test if the *Drosophila* homolog of SMN1 (called Smn) [32]–[34] functions on the same pathway as Zpr1 in tracheal terminal cell lumen formation we used RNAi to down-regulate Smn in the developing tracheal system. When we did this, we found that terminal cells lack their gas-filled lumens, a phenotype very similar to *Zpr1* loss of function (Fig. 4). This result suggests that Zpr1 functions in conjunction with Smn in terminal cell lumen formation, much as their mammalian homologs do in other biological contexts.

## Discussion

To achieve complete interconnectivity the *Drosophila* tracheal system undergoes extensive branching and fusion steps beginning in embryogenesis and continuing throughout larval development [2]. After branch outgrowth, terminal cells must then undergo an additional intracellular lumen formation step to promote gas exchange. We show here that Zpr1 is required for this lumenogenesis process: *Zpr1* mutant terminal cells show a complete absence of a gas-filled lumen. Marker and ultrastructural analysis indicates Zpr1 mutant cells can initiate terminal cell lumen formation, but the lumen cannot mature to a functional gas-filled form. *Zpr1* mutant terminal cells also show significant branching and outgrowth defects. We do not know if these defects are related to the lumenogenesis defect or represent a different aspect of Zpr1 function.

Zpr1 was initially identified as a protein that was able to bind the intracellular tail of the inactive EGFR, an interaction necessary for cell growth and division [35], [36]. Although the EGFR has previously been shown to be involved early in tracheal cell migration [23], [37], and is also necessary for maintaining the structural integrity of the embryonic tracheal system [24], specific downstream effectors have not been identified in this tissue. Recently, indirect evidence has suggested a role for the EGFR in tracheal lumen formation [22]. This work involved studying receptor protein tyrosine phosphatases (RPTP) which are negative regulators of RTK signaling. Disruption of *Ptp4E* and *Ptp10D* (the only RPTPs expressed in the tracheal system) results in a cystic lumen or luminal overgrowth phenotype in the embryonic tracheal system [22]. Reduction of EGFR activity in the *Ptp4E Ptp10D* mutant background ameliorated this defect suggesting it is over activity of this receptor that is responsible for the excessive lumen phenotype. However, the effect of direct removal of EGFR on lumen formation was not tested.

Our results show that the EGFR is required not only for embryonic tracheal lumen formation, but also for postembryonic subcellular lumenogenesis. However, our loss-of-function experiments suggest the situation is more complex than a simple requirement of the EGFR for lumenogenesis. We find that while loss of EGFR activity can lead to defects in lumenogenesis, this defect is not completely penetrant; only ∼15% of mutant cells show the defect. We do not think that this lack of penetrance is due to perdurance of EGFR gene product in the mutant cells, since we observe very similar defects in which the EGFR activity has been reduced using RNAi, which should target EGFR mRNA, and EGFR dominant negative, which should directly target EGFR protein. Rather, we think that additional RTKs or other signaling pathways are acting redundantly with the EGFR in the lumen formation process.

One candidate RTK that could be acting redundantly with EGFR is the FGFR, Btl. Btl is known to function during terminal cell development [38], where it stimulates branch outgrowth towards hypoxic tissue [11]. We cannot test the role of Btl directly, since it is required for branching outgrowth, both during embryonic and larval tracheal cell development, but we show here that ectopic Btl signaling leads to extra lumen growth, similar to what has been observed using overexpression of the Btl FGF ligand *branchless* [11]. Furthermore, we show that Zpr1 is required for both EGFR- and FGFR-stimulated lumen formation.

The finding that Zpr1 lies downstream of multiple RTKs suggests a model in which terminal cells use multiple signaling pathways to stimulate lumen formation or maturation. One possibility for these semi-redundant pathways is that they are used to interpret positional cues which help the cell determine whether it has reached hypoxic target tissue. Hypoxic tissue secretes FGF as a long-range signaling molecule that guides the outgrowth and active migration of terminal cell branches towards hypoxic tissue [11]. In our model (Fig. 4), hypoxic tissue additionally secretes EGF as a short-range signaling molecule that induces lumen formation when the terminal cell branch contacts the hypoxic tissue. Lumen formation is an important step in facilitating gas exchange to hypoxic tissue, so cross talk between the FGFR and EGFR signaling cascades provide the cell with redundant mechanisms ensuring lumen formation occurs at an appropriate time and place, as well as a mechanism for modifying the amount of lumenogenesis that occurs during hypoxic conditions. Loss of EGFR can be compensated for by increased FGF signaling, thus appearing as a lack of phenotypic penetrance when EGF signaling is reduced.

**Figure 5:**
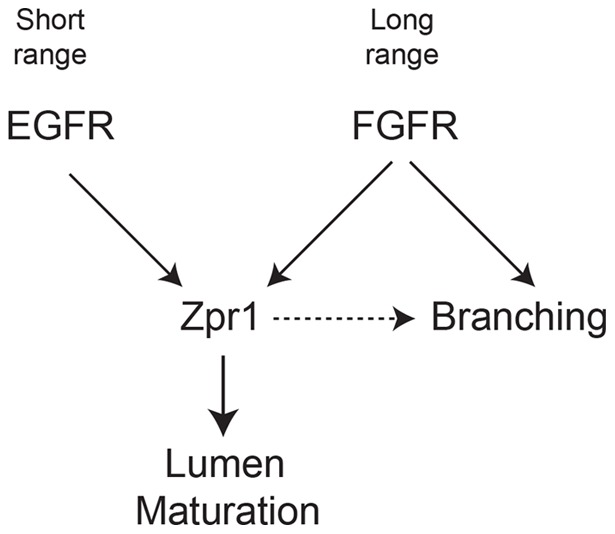
Model of Zpr1 function in terminal cell lumen maturation. FGF is a long-range signal required for outgrowth and branching in terminal cells. Short-range EGF signaling through Zpr1 initiates lumen maturation in terminal branches that have arrived at hypoxic tissue. FGF signaling can also stimulate lumen formation through Zpr1, providing redundancy in the lumenogenesis program. Zpr1 may also promote FGF-mediated branch outgrowth (dashed arrow).

We further propose Zpr1 functions to integrate the EGF and FGF signal in triggering lumen maturation (Fig. 5). *Zpr1* loss of function leads to non-gas filled cells and is epistatic to disregulated RTK signaling. Zpr1 was first identified as binding to the intracellular domain of the EGFR [16], and this is to our knowledge the first *in vivo* evidence that Zpr1 functions downstream of the EGFR in a specific developmental process. Experiments aimed at mapping the EGFR-Zpr1 binding domains showed that Zpr1 binds to the subdomains X and XI of the EGFR [16]. This region is composed of three α helices and is well conserved in other RTKs, such as the platelet-derived growth factor (PDGF) and the FGFR [39]. The binding of Zpr1 to this conserved motif on different RTKs was confirmed for the PDGF [16]; interaction with the FGFR has not been directly tested. Although the degree of conservation in subdomains X and XI of the FGFR suggests that the downstream function of Zpr1 could be mediated through their physical interaction, we have yet to test this hypothesis through co-immunoprecipitation (Co-IP) or similar experiments.

What is the mechanism by which Zpr1 stimulates lumen maturation? It is known that after release from the EGFR, Zpr1 can form complexes with the survival motor neurons 1 (SMN1) protein [17]. We show here that the *Drosophila* homolog of SMN1, Smn [32], is required for lumen development in terminal cells, much like Zpr1. The Zpr1/SMN1 interaction is known to lead to relocalization of Zpr1 to the cell nucleus [30], where it could have a role in influencing gene expression that could include a lumenogenic program. Alternatively, Zpr1 is also known to interact with the eukaryotic translation elongation factor 1α (EF1α) [40] and it has been shown that EF1α possesses microtubule severing activity [41]. Microtubules have recently been implicated in terminal cell lumenogenesis [20], suggesting a mechanism by which Zpr1 could act directly in the cytoplasm to regulate cytoskeletal components that maybe integral to lumen formation or maturation. Further experiments on the RTK/Zpr1 signaling pathway should reveal if either, or both, of these processes are required for *in vivo* subcellular lumen formation.

## Materials and Methods

### Fly stocks and genetics

All flies were reared on standard media and raised at 25°C. Control chromosomes used for clonal analysis were *FRT^19A^* [42] or *FRT^G13^* [43]. MARCM analysis [18] was done on the following alleles, *Zpr1^31ZZ^* (this work), *EGFR^K05115^* [44], *EGFR^f2^* [45], and *EGFR^top-co^* [46] (a gift from M. Fuller). The *btl-GAL4* driver was used for all tissue-specific experiments [25]. Positively marked homozygous mutant cells were generated using the stocks: *y w P{w^+^, btl-Gal80}, FRT^19A^, hsFLP^122^; btl-Gal4, UAS-GFP* and *y w hsFLP^122^; FRT^G13^, P{w^+^, tub-Gal80}; btl-Gal4, UAS-GFP* (a gift from S. Luschnig). The following EGFR RNAi transgenic lines were used: *y^1^ v^1^; P{TRiP.JF01368}attP2* and *y^1^ v^1^; P{TRiP.JF01084}attP2* and *y^1^ v^1^; P{TRiP.JF02384}attP2*. For dominant negative experiments, we used y^1^ w*; P{UAS-Egfr.DN.B}29-77-1; P{UAS-Egfr.DN.B}29-8-1 [47]. For all RTK epistasis experiments a *y w P{w^+^ tub-Gal80}, FRT^19A^, hsFLP^122^*; *btl-Gal4, UAS-DsRed* stock was used to generate MARCM clones that were homozygous mutant for *Zpr1.* The following transgenes were used for overexpression experiments: w*; P{w[+mC] = Egfr.2.UAS}7-10 and w*; P{w[+mC] = Egfr.2.A887T.UAS}8-2 [48] and *UAS-λbtl* [27]. The presence or absence of these transgenes in the MARCM mutants was determined by scoring the presence of either of the following YFP-expressing balancer chromosomes: w*; ry^506^ Dr^1^/TM3, P{Dfd-GMR-nvYFP}3, Sb^1^ and w*; sna^Sco^/CyO, P{Dfd-GMR-nvYFP} [49]. For *Smn* RNAi, we used line 16725R-2 from the National Institute of Genetics Fly Stock Center, Japan.

### Identification of mutation in line *31ZZ*

The lethality associated with mutant line *31ZZ* was first mapped by crossing the lethal chromosome to an X chromosome carrying multiple visible markers (*sc cv ct v g^2^ f FRT^19A^).* Trans-heterozygous F1 females were mated to males of genotype *Df(1)64c18, g^1^ sd^1^/Dp(1;2;Y)w^+^*. Viable hemizygous male progeny from this cross were scored for the visible markers, placing the *31ZZ* lethal mutation in the genetic interval between the *ct* and *v* genes, an approximately 3.2 Mb region. To further refine the location of the causative mutation, we collected viable male flies that had undergone a recombination event between the *ct* and *v* genes. The position of the recombination event was then determined by using single nucleotide polymorphisms (SNPs) which differed between the lethal containing chromosome and the multiply-marked chromosome. To identify these SNPs we first designed approximately 1000 bp PCR amplicons (using Primer 3 [50]) within intergenic regions spanning the *ct*-*v* interval. We determined the sequence of amplicons generated from the parental chromosome, on which we induced *31ZZ*, and the marker chromosomes. The sequences where then compared for differences including SNPs and InDels. We next checked if the identified polymorphisms altered restriction digest enzyme sites in the amplicons, and if so we used restriction digest of amplicons for genotyping. For SNPs identified in which restriction digest enzyme sites were not altered, we used sequencing to type the markers. A total of 38 recombinant flies were collected representing reciprocal recombination classes (8 *ct v* ^+^ and 30 *ct^+^v*) and tested for our identified SNPs. This analysis allowed us to narrow the genetic interval to a ∼115 kb region (between the SNPs XD11 and XA37). To further refine the position of the lethal mutation we mapped the position of the lethal mutation with respect to transposable element (TE) insertions. We obtained TE lines whose genomic location had also been mapped to between XD11 and XA37, and crossed *31ZZ* heterozygous females to males from the individual TE lines. We then crossed approximately 400 *trans*-heterozygous F1 females to males, and scored viable male progeny for the absence of visible markers (*w^+^* or *y^+^*) present on the TE, thus selecting for recombination events between the lethal mutation and the TE. By scoring flanking genetic markers, this allowed us to map the position of lethal mutation as being to the left or right of each individual TE insertion. By combining our SNP mapping data with our TE mapping results we were able to narrow the *31ZZ* genetic interval from the ∼115 kb region to a ∼72 kb region (between TE insertion *PBac{WH}-f02398* [51] and SNP XA37). All genes lying within this genetic interval were then examined for mutations by sequencing *trans*-heterozygous females carrying the *31ZZ* chromosome over a wild-type chromosome. From this, a single, non-synonymous mutation was identified in *Zinc finger protein 1* (*Zpr1*).

### Transgenes and cloning

The 3.2 kb rescue transgene spanning the *Zpr1* genomic locus and upstreamregion was amplified by PCR and cloned into the *Not*I and *Pme*I restriction enzyme sites in the P [acman] transformation vector [52] and injected into flies carrying the VK00026 attP docking site on the 3^rd^ chromosome for site-specific integration [52]. We obtained a plasmid containing *Drosophila Zpr1* cDNA (LD37736, Gold Collection) from the *Drosophila* Genomics Resource Center and PCR amplified the coding sequence for subsequent cloning. The coding sequence of *Zpr1* was then cloned into the pUAST+attB (a gift from Carl Thummel) transformation vector using the *Not*I and *EcoR*V restriction sites and injected into flies carrying the VK00027 attP docking site on the 3^rd^ chromosome. We constructed a *Zpr1* RNAi transgene by cloning two copies of a 450 bp amplicon derived from the *Zpr1* coding sequence (bases 88–537) in reverse tandem orientation around the intron in the pUASTi vector [15]. Flies were transformed with this construct using standard P-element mediated transgenesis. All transgenic injections were done by Genetic Services Inc. (Cambridge, MA).

### Terminal cell branching and lumen quantification

To determine the number of branches in wild-type and mutant cells, we collected fluorescence images of GFP-labeled dorsal terminal cells. Branches from these cells were traced manually using the NeuronJ plug-in for ImageJ [53]. Branch order was assigned by the following scheme: the single central branch encompassing the nucleus was designated as the primary branch of each cell; secondary branches arise directly from that primary branch; branches that arise from secondary branches were categorized as tertiary branches, which in turn give rise to quaternary branches. To assay lumen formation in terminal cells, we used brightfield optics to visualize gas-filling. Since mutants were either completely gas-filled or completely empty and we did not see partial gas-filling we used a binary scoring system in which individual cells were scored as either wild-type or defective for gas-filling. In experiments using tracheal-specific RNAi or dominant-negative transgenes we scored the lateral terminal cells in the Tr 3 and 4 tracheal hemisegments of individual animals.

### Antibody staining

We used α-Wkdpep antisera [20] diluted 1∶500 and goat anti-rabbit conjugated to Alexa-568 secondary diluted 1∶1000 to stain L3 larval filets, as previously described [54].

### Electron microcopy methods

Full details of the EM methods will be published elsewhere (L.N. & M.M.M, in prep). Briefly, we prepared late L1 or early L2 wild-type or *Zpr1* RNAi larvae for EM using a high pressure freezing/freeze substitution approach [55]. Frozen larvae were fixed with 1% osmium tetroxide (OsO_4_) with 0.1% Uranyl Acetate in 97% acetone. Fixed samples were embedded in Durcupan resin, sectioned, and imaged at 125keV using a Hitachi 7200 electron microscope.

## Supporting Information

Figure S1 **TEM analysis of** ***Zpr1*** **mutant terminal cells.** GFP (A) and brightfield (A') images of terminal cells expressing RNAi directed against *Zpr1*. (B) Further examples of TEM analysis of terminal cell branches in which *Zpr1* has been inactivated by RNAi. The lumens (arrows) are occluded and lack a mature chitinous lining. Bars, 400 nm.
(TIF)

## References

[1] Ghabrial A, Luschnig S, Metzstein MM, Krasnow MA (2003) Branching morphogenesis of the Drosophila tracheal system. Annu Rev Cell Dev Biol 19: 623–647 doi:10.1146/annurev.cellbio.19.031403.160043. 14570584 10.1146/annurev.cellbio.19.031403.160043

[2] Affolter M, Caussinus E (2008) Tracheal branching morphogenesis in Drosophila: new insights into cell behaviour and organ architecture. Development (Cambridge, England) 135: 2055–2064 doi:10.1242/dev.014498. 10.1242/dev.014498 18480161

[3] Sutherland D, Samakovlis C, Krasnow MA (1996) branchless encodes a Drosophila FGF homolog that controls tracheal cell migration and the pattern of branching. Cell 87: 1091–1101. 8978613 10.1016/s0092-8674(00)81803-6

[4] Klämbt C, Glazer L, Shilo BZ (1992) breathless, a Drosophila FGF receptor homolog, is essential for migration of tracheal and specific midline glial cells. Genes & development 6: 1668–1678. 1325393 10.1101/gad.6.9.1668

[5] Samakovlis C, Hacohen N, Manning G, Sutherland DC, Guillemin K, et al (1996) Development of the Drosophila tracheal system occurs by a series of morphologically distinct but genetically coupled branching events. Development (Cambridge, England) 122: 1395–1407. 10.1242/dev.122.5.1395 8625828

[6] Shim K, Blake KJ, Jack J, Krasnow MA (2001) The Drosophila ribbon gene encodes a nuclear BTB domain protein that promotes epithelial migration and morphogenesis. Development (Cambridge, England) 128: 4923–4933. 10.1242/dev.128.23.4923 11731471

[7] Bradley PL, Andrew DJ (2001) ribbon encodes a novel BTB/POZ protein required for directed cell migration in Drosophila melanogaster. Development (Cambridge, England) 128: 3001–3015. 10.1242/dev.128.15.3001 11532922

[8] Ribeiro C, Neumann M, Affolter M (2004) Genetic Control of Cell Intercalation during Tracheal Morphogenesis in Drosophila. Current Biology 14: 2197–2207 doi:10.1016/j.cub.2004.11.056. 15620646 10.1016/j.cub.2004.11.056

[9] Guillemin K, Groppe J, Ducker K, Treisman R, Hafen E, et al (1996) The pruned gene encodes the Drosophila serum response factor and regulates cytoplasmic outgrowth during terminal branching of the tracheal system. Development (Cambridge, England) 122: 1353–1362. Available:http://eutils.ncbi.nlm.nih.gov/entrez/eutils/elink.fcgi?dbfrom=pubmed&id=8625824&retmode=ref&cmd=prlinks. 10.1242/dev.122.5.1353 8625824

[10] Gervais L, Casanova J (2010) In vivo coupling of cell elongation and lumen formation in a single cell. Curr Biol 20: 359–366 doi:10.1016/j.cub.2009.12.043. 20137948 10.1016/j.cub.2009.12.043

[11] Jarecki J, Johnson E, Krasnow MA (1999) Oxygen regulation of airway branching in Drosophila is mediated by branchless FGF. Cell 99: 211–20. 10535739 10.1016/s0092-8674(00)81652-9

[12] Centanin L, Dekanty A, Romero N, Irisarri M, Gorr TA, et al (2008) Cell autonomy of HIF effects in Drosophila: tracheal cells sense hypoxia and induce terminal branch sprouting. Developmental Cell 14: 547–558 doi:10.1016/j.devcel.2008.01.020. 18410730 10.1016/j.devcel.2008.01.020

[13] Egginton S (2011) Physiological factors influencing capillary growth. Acta Physiologica 202: 225–239 doi:10.1111/j.1748–1716.2010.02194.x. 20946238 10.1111/j.1748-1716.2010.02194.x

[14] Baer MM, Bilstein A, Leptin M (2007) A clonal genetic screen for mutants causing defects in larval tracheal morphogenesis in Drosophila. Genetics 176: 2279–2291 doi:10.1534/genetics.107.074088. 17603107 10.1534/genetics.107.074088 PMC1950631

[15] Ghabrial AS, Levi BP, Krasnow MA (2011) A systematic screen for tube morphogenesis and branching genes in the Drosophila tracheal system. PLoS Genet 7: e1002087 doi:10.1371/journal.pgen.1002087. 21750678 10.1371/journal.pgen.1002087 PMC3131284

[16] Galcheva-Gargova Z, Konstantinov KN, Wu IH, Klier FG, Barrett T, et al (1996) Binding of zinc finger protein ZPR1 to the epidermal growth factor receptor. Science (New York, NY 272: 1797–1802. 10.1126/science.272.5269.1797 8650580

[17] Gangwani L, Mikrut M, Theroux S, Sharma M, Davis RJ (2001) Spinal muscular atrophy disrupts the interaction of ZPR1 with the SMN protein. Nat Cell Biol 3: 376–383 doi:10.1038/35070059. 11283611 10.1038/35070059

[18] Lee T, Luo L (1999) Mosaic analysis with a repressible cell marker for studies of gene function in neuronal morphogenesis. Neuron 22: 451–461. 10197526 10.1016/s0896-6273(00)80701-1

[19] Brand AH, Manoukian AS, Perrimon N (1994) Ectopic expression in Drosophila. Methods in cell biology 44: 635–654. 7707973 10.1016/s0091-679x(08)60936-x

[20] Schottenfeld-Roames J, Ghabrial AS (2012) Whacked and Rab35 polarize dynein-motor-complex-dependent seamless tube growth. Nat Cell Biol 14: 386–393 doi:10.1038/ncb2454. 22407366 10.1038/ncb2454 PMC3334817

[21] Wilk R, Reed BH, Tepass U, Lipshitz HD (2000) The hindsight Gene Is Required for Epithelial Maintenance and Differentiation of the Tracheal System in Drosophila. Developmental biology 219: 183–196 doi:10.1006/dbio.2000.9619. 10694415 10.1006/dbio.2000.9619

[22] Jeon M, Zinn K (2009) Receptor tyrosine phosphatases control tracheal tube geometries through negative regulation of Egfr signaling. Development (Cambridge, England) 136: 3121–3129 doi:10.1242/dev.033597. 10.1242/dev.033597 PMC2730367 19675131

[23] Llimargas M, Casanova J (1999) EGF signaling regulates cell invagination as well as cell migration during formation of tracheal system in Drosophila. Dev Genes Evol 209: 174–179. 10079360 10.1007/s004270050241

[24] Cela C (2006) Egfr is essential for maintaining epithelial integrity during tracheal remodelling in Drosophila. Development (Cambridge, England) 133: 3115–3125 doi:10.1242/dev.02482. 10.1242/dev.02482 16831830

[25] Shiga Y, Tanaka-Matakatsu M, Hayashi S (1996) A nuclear GFP/ß-galactosidase fusion protein as a marker for morphogenesis in living Drosophila. Dev Growth Differ 38: 99–106.

[26] Glazer L, Shilo BZ (1991) The Drosophila FGF-R homolog is expressed in the embryonic tracheal system and appears to be required for directed tracheal cell extension. Genes & development 5: 697–705. 1849109 10.1101/gad.5.4.697

[27] Lee T, Hacohen N, Krasnow M, Montell DJ (1996) Regulated Breathless receptor tyrosine kinase activity required to pattern cell migration and branching in the Drosophila tracheal system. Genes & development 10: 2912–2921. 8918892 10.1101/gad.10.22.2912

[28] Mishra AK, Gangwani L, Davis RJ, Lambright DG (2007) Structural insights into the interaction of the evolutionarily conserved ZPR1 domain tandem with eukaryotic EF1A, receptors, and SMN complexes. Proceedings of the National Academy of Sciences of the United States of America 104: 13930–13935 doi:10.1073/pnas.0704915104. 17704259 10.1073/pnas.0704915104 PMC1955815

[29] Ahmad S, Wang Y, Shaik GM, Burghes AH, Gangwani L (2012) The zinc finger protein ZPR1 is a potential modifier of spinal muscular atrophy. Hum Mol Genet 21: 2745–2758 doi:10.1093/hmg/dds102. 22422766 10.1093/hmg/dds102 PMC3363332

[30] Gangwani L, Flavell RA, Davis RJ (2005) ZPR1 is essential for survival and is required for localization of the survival motor neurons (SMN) protein to Cajal bodies. Molecular and cellular biology 25: 2744–2756 doi:10.1128/MCB.25.7.2744–2756.2005. 15767679 10.1128/MCB.25.7.2744-2756.2005 PMC1061650

[31] Doran B, Gherbesi N, Hendricks G, Flavell RA, Davis RJ, et al (2006) Deficiency of the zinc finger protein ZPR1 causes neurodegeneration. Proceedings of the National Academy of Sciences of the United States of America 103: 7471–7475 doi:10.1073/pnas.0602057103. 16648254 10.1073/pnas.0602057103 PMC1464363

[32] Miguel-Aliaga I, Chan YB, Davies KE, van den Heuvel M (2000) Disruption of SMN function by ectopic expression of the human SMN gene in Drosophila. FEBS Lett 486: 99–102. 11113446 10.1016/s0014-5793(00)02243-2

[33] Rajendra TK, Gonsalvez GB, Walker MP, Shpargel KB, Salz HK, et al (2007) A Drosophila melanogaster model of spinal muscular atrophy reveals a function for SMN in striated muscle. The Journal of cell biology 176: 831–841 doi:10.1083/jcb.200610053. 17353360 10.1083/jcb.200610053 PMC2064057

[34] Chan YB, Miguel-Aliaga I, Franks C, Thomas N, Trülzsch B, et al (2003) Neuromuscular defects in a Drosophila survival motor neuron gene mutant. Hum Mol Genet 12: 1367–1376. 12783845 10.1093/hmg/ddg157

[35] Galcheva-Gargova Z, Gangwani L, Konstantinov KN, Mikrut M, Theroux SJ, et al (1998) The cytoplasmic zinc finger protein ZPR1 accumulates in the nucleolus of proliferating cells. Mol Biol Cell 9: 2963–2971. 9763455 10.1091/mbc.9.10.2963 PMC25573

[36] Gangwani L (2006) Deficiency of the zinc finger protein ZPR1 causes defects in transcription and cell cycle progression. J Biol Chem 281: 40330–40340 doi:10.1074/jbc.M608165200. 17068332 10.1074/jbc.M608165200

[37] Nishimura M, Inoue Y, Hayashi S (2007) A wave of EGFR signaling determines cell alignment and intercalation in the Drosophila tracheal placode. Development (Cambridge, England) 134: 4273–4282 doi:10.1242/dev.010397. 10.1242/dev.010397 17978004

[38] Reichman-Fried M, Shilo BZ (1995) Breathless, a Drosophila FGF receptor homolog, is required for the onset of tracheal cell migration and tracheole formation. Mechanisms of development 52: 265–273. Available:http://eutils.ncbi.nlm.nih.gov/entrez/eutils/elink.fcgi?dbfrom=pubmed&id=8541215&retmode=ref&cmd=prlinks. 10.1016/0925-4773(95)00407-r 8541215

[39] Hubbard SR, Wei L, Ellis L, Hendrickson WA (1994) Crystal structure of the tyrosine kinase domain of the human insulin receptor. Nature 372: 746–754 doi:10.1038/372746a0. 7997262 10.1038/372746a0

[40] Gangwani L, Mikrut M, Galcheva-Gargova Z, Davis RJ (1998) Interaction of ZPR1 with translation elongation factor-1alpha in proliferating cells. The Journal of cell biology 143: 1471–1484. 9852145 10.1083/jcb.143.6.1471 PMC2132977

[41] Shiina N, Gotoh Y, Kubomura N, Iwamatsu A, Nishida E (1994) Microtubule severing by elongation factor 1 alpha. Science (New York, NY 266: 282–285. 10.1126/science.7939665 7939665

[42] Xu T, Rubin GM (1993) Analysis of genetic mosaics in developing and adultDrosophilatissues. Development (Cambridge, England) 117: 1223–1237. 10.1242/dev.117.4.1223 8404527

[43] Chou TB, Perrimon N (1992) Use of a yeast site-specific recombinase to produce female germline chimeras in Drosophila. Genetics 131: 643–653. 1628809 10.1093/genetics/131.3.643 PMC1205036

[44] Bier E, Vaessin H, Shepherd S, Lee K, McCall K, et al (1989) Searching for pattern and mutation in the Drosophila genome with a P-lacZ vector. Genes & development 3: 1273–1287. 2558049 10.1101/gad.3.9.1273

[45] Nusslein-Volhard C, Wieschaus E, Kluding H (1984) Mutations affecting the pattern of the larval cuticle in Drosophila melanogaster. Roux's Archives of Developmental Biology 193: 267–282. 10.1007/BF00848156 28305337

[46] Clifford RJ, Schüpbach T (1989) Coordinately and differentially mutable activities of torpedo, the Drosophila melanogaster homolog of the vertebrate EGF receptor gene. Genetics 123: 771–787. 2515109 10.1093/genetics/123.4.771 PMC1203888

[47] Freeman M (1996) Reiterative use of the EGF receptor triggers differentiation of all cell types in the Drosophila eye. Cell 87: 651–660. 8929534 10.1016/s0092-8674(00)81385-9

[48] Lesokhin AM, Yu SY, Katz J, Baker NE (1999) Several levels of EGF receptor signaling during photoreceptor specification in wild-type, Ellipse, and null mutant Drosophila. Developmental biology 205: 129–144 doi:10.1006/dbio.1998.9121. 9882502 10.1006/dbio.1998.9121

[49] Le T, Liang Z, Patel H, Yu MH, Sivasubramaniam G, et al (2006) A new family of Drosophila balancer chromosomes with a w- dfd-GMR yellow fluorescent protein marker. Genetics 174: 2255–2257 doi:10.1534/genetics.106.063461. 17057238 10.1534/genetics.106.063461 PMC1698648

[50] Rozen S, Skaletsky H (2000) Primer3 on the WWW for general users and for biologist programmers. Methods in molecular biology (Clifton, NJ 132: 365–386. 10.1385/1-59259-192-2:365 10547847

[51] Thibault ST, Singer MA, Miyazaki WY, Milash B, Dompe NA, et al (2004) A complementary transposon tool kit for Drosophila melanogaster using P and piggyBac. Nat Genet 36: 283–287. 14981521 10.1038/ng1314

[52] Venken KJ, He Y, Hoskins RA, Bellen HJ (2006) P [acman]: a BAC transgenic platform for targeted insertion of large DNA fragments in D. melanogaster. Science (New York, NY 314: 1747–1751. 10.1126/science.1134426 17138868

[53] Meijering E, Jacob M, Sarria J-CF, Steiner P, Hirling H, et al (2004) Design and validation of a tool for neurite tracing and analysis in fluorescence microscopy images. Cytometry A 58: 167–176 doi:10.1002/cyto.a.20022. 15057970 10.1002/cyto.a.20022

[54] Jones TA, Metzstein MM (2011) A novel function for the PAR complex in subcellular morphogenesis of tracheal terminal cells in Drosophila melanogaster. Genetics 189: 153–164 doi:10.1534/genetics.111.130351. 21750259 10.1534/genetics.111.130351 PMC3176136

[55] Vanhecke D, Graber W, Studer D (2008) Close-to-native ultrastructural preservation by high pressure freezing. Methods in cell biology 88: 151–164 doi:10.1016/S0091–679X(08)00409-3. 18617033 10.1016/S0091-679X(08)00409-3

