# Supplementary figures and images for: Drosophila Zpr1 (Zinc Finger Protein 1) Is Required Downstream of Both EGFR And FGFR Signaling in Tracheal Subcellular Lumen Formation
Source: PLoS One. 2012 Sep 18;7(9):e45649. doi: 10.1371/journal.pone.0045649 (PMC3445489; doi:10.1371/journal.pone.0045649)

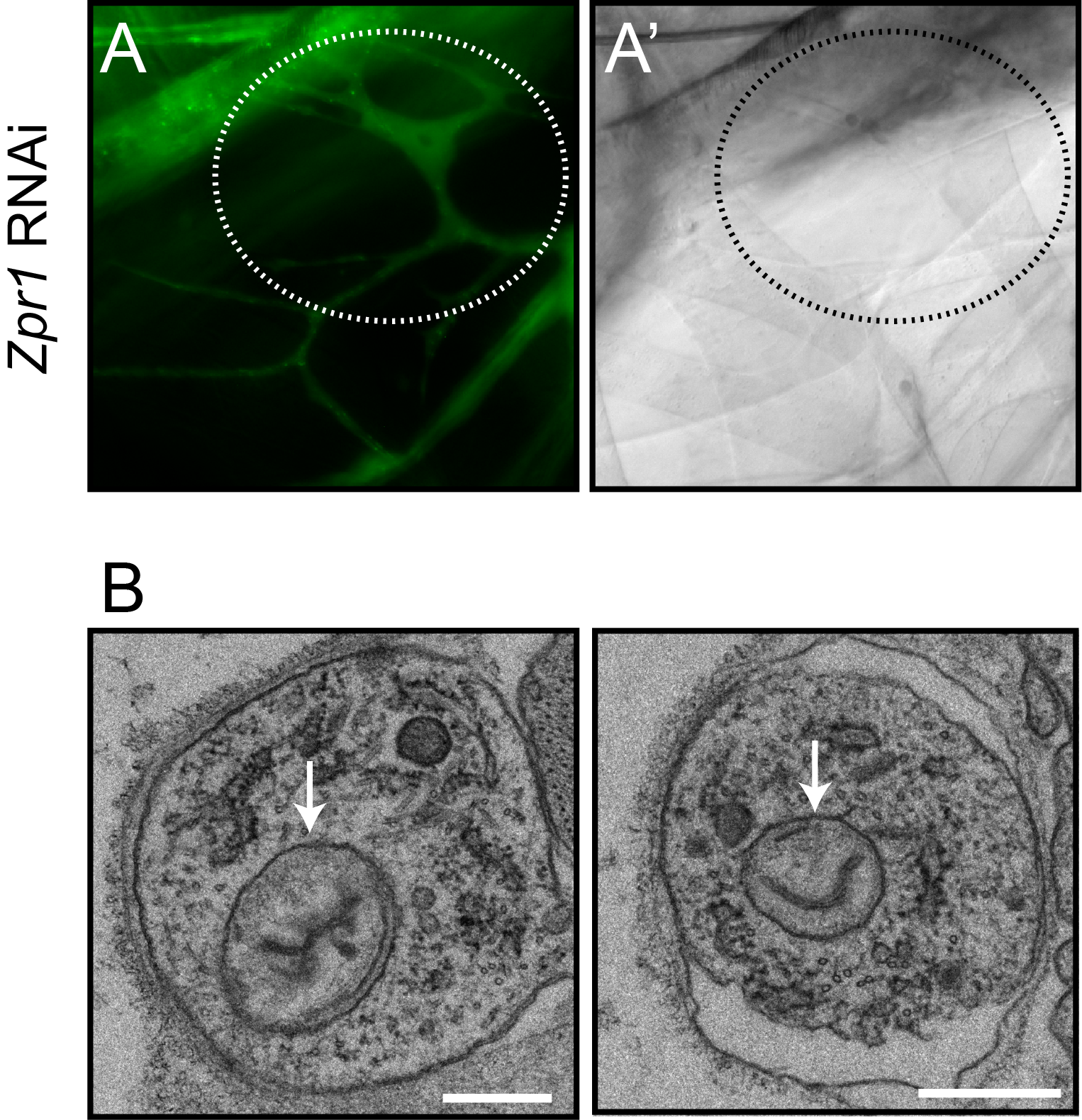

Supplement: Figure S1 — TEM analysis of Zpr1 mutant terminal cells. GFP (A) and brightfield (A') images of terminal cells expressing RNAi directed against Zpr1. (B) Further examples of TEM analysis of terminal cell branches in which Zpr1 has been inactivated by RNAi. The lumens (arrows) are occluded and lack a mature chitinous lining. Bars, 400 nm. (TIF) [file pone.0045649.s001.tif]
